# Function and mechanism of miRNAs during the process of *Klebsiella pneumoniae* infection: A review

**DOI:** 10.17305/bb.2025.11421

**Published:** 2025-02-25

**Authors:** Chuhan Zhang, Ge Li, Safi Ullah, Liang Liu, Huajie Zhao, Fan Yang, Liwei Guo, Duan Li

**Affiliations:** 1 Department of Pathogenic Biology, School of Basic Medicine, Xinxiang Medical University, Xinxiang, Henan, China; 2 School of Basic Medicine, Xinxiang Medical University, Xinxiang, Henan, China; 3 School of International Education, Xinxiang Medical University, Xinxiang, Henan, China; 4 School of Forensic Medicine, Xinxiang Medical University, Xinxiang, Henan, China

**Keywords:** *Klebsiella pneumoniae*, miRNAs, lung infection, peritonitis, sepsis

## Abstract

*Klebsiella pneumoniae* (*K. pneumoniae*), a Gram-negative bacterium, is a major cause of nosocomial infections and can lead to severe, widespread infections. The rise of hypervirulent and multidrug-resistant *K. pneumoniae* presents significant challenges to public health. Diseases associated with *K. pneumoniae*, such as pneumonia, lung injury, peritonitis, and sepsis, have garnered increasing attention. MicroRNAs (miRNAs) are a class of short, endogenously expressed non-coding RNAs that regulate gene expression by inhibiting translation or promoting mRNA degradation. As key regulators of gene expression, miRNAs play a crucial role in *K. pneumoniae* infections by modulating host inflammatory pathways, suppressing inflammasome activity, regulating cytokine secretion, and facilitating post-translational modifications. Understanding miRNA alterations and their mechanisms during *K. pneumoniae* infections is of great significance. This comprehensive review explores the functions and mechanisms of miRNAs in *K. pneumoniae*-induced lung injury, peritonitis, and sepsis. By analyzing differential miRNA expression during infection, we aim to provide new insights and potential directions for future clinical diagnosis and treatment strategies for *K. pneumoniae* infections.

## Introduction

**Figure 1.**
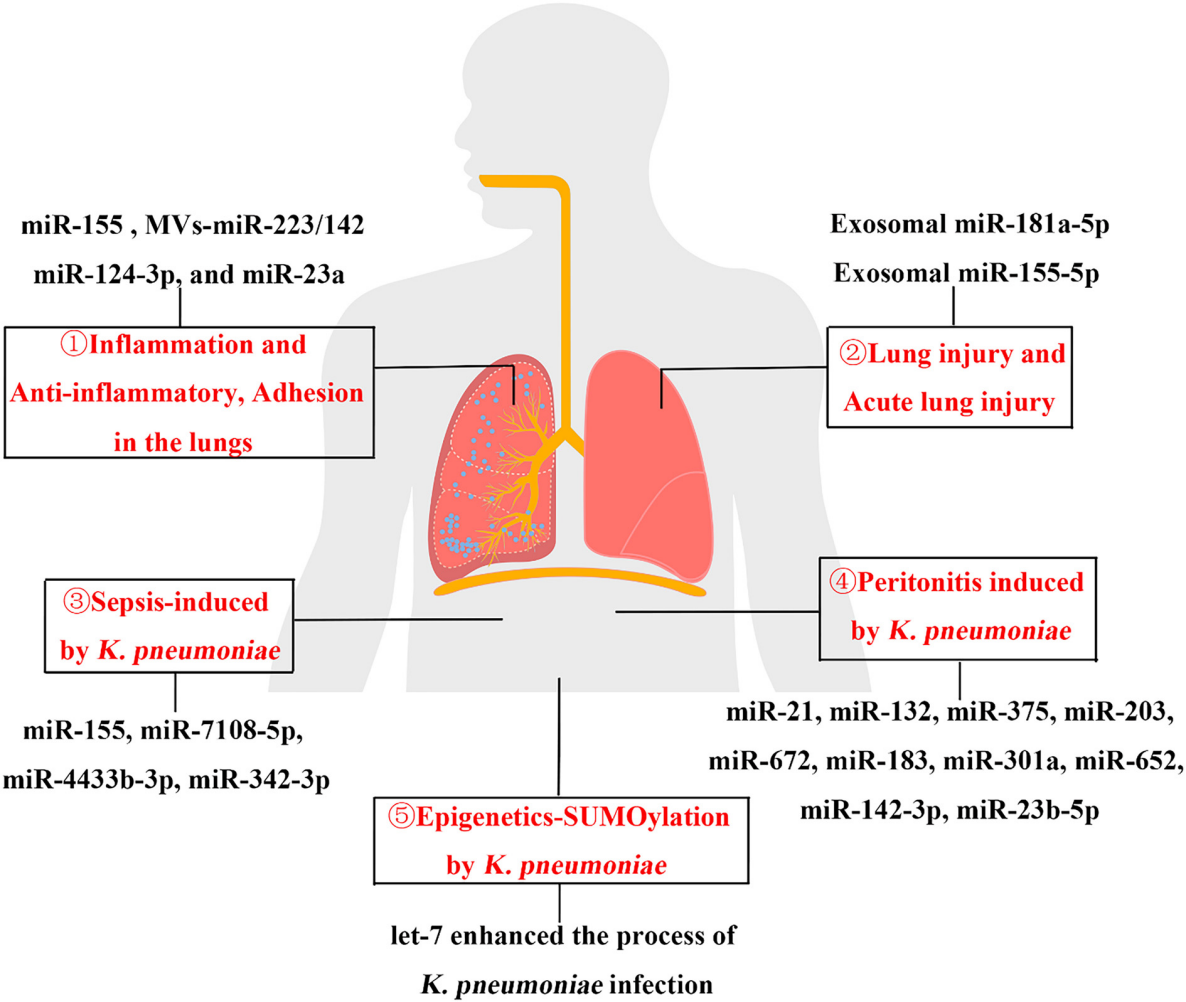
**Image summary of the profile of miRNAs involved in *K. pneumoniae* infection.** Diseases caused by *K. pneumoniae* infection and the profile of associated miRNAs. Red Arabic numbers with circles represent lung diseases (① and ②), systemic sepsis (③), abdominal peritonitis (④) and epigenetics (⑤) caused by *Klebsiella pneumoniae* infection. miRNA: MicroRNA.

*Klebsiella pneumoniae* (*K. pneumoniae*) was first described as a bacterium isolated from the lungs of patients who had died from pneumonia. It was later found on the mucosal surfaces of the oropharynx, nasopharynx, upper respiratory tract, and gastrointestinal tract in patients [1–3]. *K. pneumoniae* can cause various diseases, including pneumonia, sepsis, and urinary tract infections [4]. Virulence factors, such as capsules, lipopolysaccharides, membranes, and iron-acquisition systems play a crucial role in the pathogenicity of *K. pneumoniae* [5]. These factors contribute significantly to adherence, colonization, invasion, and disease progression. There are two major variants of *K. pneumoniae*: classical *K. pneumoniae* (cKp) and hypervirulent *K. pneumoniae* (hvKp) [6]. In recent years, a novel classification system has been proposed to distinguish ultravirulent and supervirulent strains from both cKp and hvKp [7]. Traditionally, cKp has been the most common form of *K. pneumoniae* in Western countries. However, through the acquisition of virulence factors encoded on plasmids and mobile genetic elements, it has evolved into a more aggressive pathogen [6]. Furthermore, the emergence and spread of multidrug-resistant *K. pneumoniae* (MDR-Kp), including carbapenem-resistant strains (CR-Kp), pose significant challenges to antibiotic treatment, leading to severe infections and high mortality rates [8]. As a result, extensive research has been conducted to better understand the dissemination of resistance genes between different *K. pneumoniae* clones, which can give rise to more pathogenic multidrug-resistant strains [9, 10]. MicroRNAs (miRNAs) are small RNA molecules, approximately 22 nucleotides in length, that regulate gene expression by binding to complementary regions in the 3′ untranslated region (3′ UTR) of target mRNAs, leading to either transcriptional degradation or translation inhibition [11, 12]. miRNAs can modulate entire cellular signaling pathways, restoring cellular functions altered by disease [13]. They play a key role in various biological processes, including developmental timing, host-pathogen interactions, cell differentiation, proliferation, apoptosis, and tumorigenesis [14]. Some miRNAs enhance the host immune response during bacterial infections while also mitigating inflammation-related damage [15]. Growing evidence suggests that miRNAs play a crucial regulatory role in lung and other organ diseases caused by *K. pneumoniae* infection [16–18], as well as in cancer [19]. This article reviews the major diseases caused by *K. pneumoniae* infection and their associated miRNAs, as illustrated in Figure 1.

### miRNAs participating in *K. pneumoniae* infection

Several studies have reported differential expression of specific miRNAs in diseases induced by *K. pneumoniae* infection. miR-124-3p and exosomal miR-155-5p (detailed mechanism in Table 1) regulate the p38 mitogen-activated protein kinase (p38-MAPK) signaling pathway, thereby enhancing the inflammatory response triggered by *K. pneumoniae* infection [16, 20]. Multiple miRNAs interact to influence Toll-like receptor (TLR) signaling and contribute to the recognition of *K. pneumoniae*. For instance, miR-146a targets key components of the TLR/interleukin-1 (IL-1) receptor pathway, including IL-1 receptor-associated kinase 1/2 (IRAK1/2) and tumor necrosis factor α (TNFα) receptor-associated factor (TRAF) [21]. Additionally, the TLR signaling pathway can regulate miRNA expression during *K. pneumoniae* infection. A notable example is the modulation of let-7 expression via the TLR4-TRAM-TRIF signaling pathway [22].

**Table 1:** Regulation of miRNAs in *K. pneumoniae* infection

**miRNA**	**Model**	**Differential expression**	**Relevant target or involved signaling pathway**	**Biological function**	**Ref.**	**Year**
miR-124 -3p	Rat lung tissue	down	p38 and p38MAPK signali ng pathway	Play anti-inflammatory function, improve lung injury	[16]	2023
miR-23a	A549	down	HMGN2 and the integrin α5β1/Rac pathway	Regulate the adhesion of K. pneumoniae to human lung epithelial cells	[31]	2016
miR-155	A549	down	HMGN2, NFI and the integrin α5β1/Rac pathway	Regulate the adhesion of K. pneumoniae to human lung epithelial cells	[31]	2016
exo-miR-155	RAW264.7	up	MSK1 and MSK1/DUSP1/p38-MAPK pathway	Induce macrophage M1 polarization and inflammatory response, enhance K. pneumoniae sepsis- associated acute lung injury	[20]	2023
miR-181 a-5p	BALF, BMDM	up	NLRP3, ASC and STAT3 signaling pathway	Alleviates the effects of lung damage induced by K. pneumoniae infection	[17]	2022
MV-miR- 223/142	BALF	up	NLRP and ASC	Significant anti-inflammatory effect on lung	[18]	2019
miR-155	PECs	down	SOCS1, SHIP1 and TLR signaling pathway	Negatively regulate TLR pathway and play an anti-inflammatory role	[21]	2013
miR-146a	PECs	up	IRAK 1/2 TRAF 6 and TLR/IL-1 receptor pathway	Anti-inflammatory role	[21]	2013
miR-142-3p, -146a, -299 and -200c	PECs	up	mRNA and protein levels of HMGB1	Modulates host inflammatory response	[21]	2013
miR-132	PECs	up	IL-1β	Associated with the development of tolerance to K. pneumoniae	[21]	2013
miR-21, miR-142-3p	PECs	up	IL-6	Associated with the development of tolerance to K. pneumoniae	[21]	2013
Let-7	Macrophage	up	TLR4-TR AM-TRIF-IFN-IFNAR1	Inhibit SUMOylation and promote K. pneumoniae infection and inflammation	[22]	2020

K. pneumonia: Klebsiella pneumonia; miRNA: MicroRNA; SUMO: Small ubiquitin-like modifier; TLR: Toll-like receptor; STAT: Signal transducer and activator of transcription 3; p38MAPK: p38 mitogen-activated protein kinase; HMGN2: High-mobility group nucleosomal binding domain 2; NFI: Nuclear factor I; IL-1: Interleukin-1; IRAK1/2: IL-1 receptor-associated kinase 1/2; MSK1: Mitogen- and stress-activated protein kinase-1; DUSP1: Dual-specific phosphatase 1.

A variety of pro-inflammatory factors work together as key strategies of the host defense against *K. pneumoniae* infection. Among these, IL-1β, IL-6, and TNF-α—regulated by miRNAs—are well-known pro-inflammatory cytokines that serve as powerful “weapons” against various pathogens [23]. miR-181a-5p reduces the levels of IL-1β, IL-18, IL-8, TNF-α, and transforming growth factor-β (TGF-β), demonstrating a significant anti-inflammatory effect in lung tissue infected with *K. pneumoniae* [17]. In a novel chronic peritonitis model of intraperitoneal *K. pneumoniae* infection, miR-132 is predicted to target IL-1β, while upregulated miR-21 may decrease IL-6 production and lower IL-1β levels [21]. Additionally, miR-142-3p is associated with the consecutive downregulation of IL-6 and may contribute to the observed tolerance pattern [21]. The NACHT, LRR, and PYD domains-containing NLRP3, along with apoptosis-associated speck-like protein (ASC), may directly or indirectly influence the inflammatory response induced by *K. pneumoniae* infection [24]. NLRP3 has been identified as a target of miR-181a-5p and miR-223, while ASC levels are modulated by miR-181a-5p and miR-142 [17, 18]. These findings suggest a potential strategy for using specific miRNAs to regulate inflammation caused by *K. pneumoniae* infection. Furthermore, EVs are membranous structures [25], and the host may form a specialized EV-miRNA complex [18, 20] to combat *K. pneumoniae* infection [22]. These insights highlight the feasibility and potential of miRNA-based approaches for diagnosing and treating *K. pneumoniae* infections.

## Role of miRNA in pulmonary disease caused by *K. pneumoniae* infection

### miR-155 and miR-23a regulate the adhesion process of *K. pneumoniae*

miR-155 is involved in the production of pro-inflammatory cytokines and is considered a potential biomarker for various neurological diseases [26]. Additionally, miR-155 regulates the biological functions of immune cells and plays a key role in the host immune response [27]. Numerous studies have shown that miR-155 is often overexpressed during bacterial infections [28–30]. However, one study found that the expression of miR-155 and miR-23a was downregulated in pulmonary epithelial cells infected with *K. pneumoniae*. Moreover, miR-155 expression remained suppressed in RAW264.7 and A549 cells treated with LPS [31]. The same study demonstrated that high-mobility group nucleosomal binding domain 2 (HMGN2) is a target of miR-155 and miR-23a, playing a role in the adhesion process of *K. pneumoniae* [31]. Further research revealed that the integrin α5β1/Rac1 pathway and actin polymerization can partially inhibit *K. pneumoniae* adhesion, a process in which miR-155 and miR-23a are involved [31]. Overall, HMGN2 functions as an inhibitor, regulating miR-155-mediated integrin α5β1 activity in A549 cells infected with *K. pneumoniae* [31]. Interestingly, the study found that miR-155 is more dependent on the integrin α5β1/Rac1 pathway than miR-23a. Additionally, the integrin transcription suppressor nuclear factor I (NFI) is a target gene of miR-155. miR-155 regulates integrin gene function by inhibiting NFI expression during *K. pneumoniae* infection [32, 33]. In summary, the proposed mechanism of miR-155/miR-23a involvement in *K. pneumoniae* infection suggests that host cells actively suppress miR-155 and miR-23a expression. This suppression releases HMGN2 and NFI activity, which in turn significantly inhibits the activation of the integrin α5β1/Rac1 pathway and the actin cytoskeletal rearrangement required for *K. pneumoniae* adhesion.

### miR-124-3p play anti-inflammatory role in lung injury induced by *K. pneumoniae* infection

miR-124-3p disorders affect various disease characteristics [26]. Studies have shown that miR-124-3p acts as a protective agent, contributing to the anti-inflammatory process in the lungs and helping to alleviate lung injuries [34]. Mechanistically, miR-124-3p directly targets p65, reducing inflammation and pulmonary injury in a mouse model of acute respiratory distress syndrome (ARDS) [34]. As one of the most well-studied classical inflammatory pathways [35], the p38MAPK pathway plays a crucial role in inflammation [36]. Studies have shown that its phosphorylation levels increase significantly in *K. pneumoniae*-infected lung cells [37]. Chlorogenic acid, known for its anti-inflammatory properties [38], has been found to upregulate miR-124-3p expression, thereby inhibiting p38 expression and inactivating the p38MAPK pathway [16]. This suggests a potential anti-inflammatory treatment for *K. pneumoniae*-induced diseases through the chlorogenic acid/miR-124-3p/p38MAPK axis.

### miR-155 and MVs-miR-223/142 regulate pulmonary inflammation induced by *K. pneumoniae* infection

Macrophage inflammatory responses are known to promote the expression of miR-155 [39]. Previous studies have shown that miR-155 plays a crucial role in the development of immune cells [40–42]. However, in an experiment involving mice infected with *Klebsiella pneumoniae*, myeloid miR-155 deficiency did not affect the myeloid cell population in the alveolar cavity or blood, nor did it significantly regulate immune cell development. Additionally, bacterial counts in lung tissue, blood, liver, and spleen, as well as IL-6 and TNF levels in bronchoalveolar lavage fluid (BALF), were measured. The results indicated that myeloid miR-155 deficiency did not impact immune defense or inflammatory regulation during *K. pneumoniae* infection [43]. In other words, myeloid miR-155 plays a minimal role in *K. pneumoniae*- or LPS-induced pneumonia. However, further research is needed to fully understand the role of miR-155 in the inflammatory response triggered by *K. pneumoniae* infection. miR-223 and miR-142, known to be specific to hematopoietic tissues [44], are also key regulators of host inflammatory responses [45–48]. The miR-223/miR-142 pathway plays a crucial role in cell proliferation, differentiation, and development [49]. EVs are classified into MVs, exosomes, and apoptotic bodies [50]. A study analyzing BALF and serum from LPS- or *K. pneumoniae*-infected mice showed that MVs-miR-223/miR-142 secretion was significantly induced, leading to notable pulmonary anti-inflammatory effects [18]. miRNA 3′-end uridylation facilitates the packaging of miR-223/miR-142 into MVs, thereby enhancing the pulmonary inflammatory response to *K. pneumoniae* infection [18]. As key regulators of host anti-inflammatory activity, miR-223 and miR-142 inhibit the activation of the NLRP3 inflammasome in macrophages by suppressing NLRP3 and apoptosis-associated speck-like protein containing a CARD (ASC), respectively [18]. In summary, MVs-miR-223/miR-142 expression is significantly upregulated in response to LPS and *K. pneumoniae* infection (mechanisms detailed in Table 1). This study highlights the potential of MVs-miR-223/miR-142 as a promising biomarker for pulmonary inflammation induced by *K. pneumoniae* infection.

### Exosomal miR-155-5p participated in acute lung injury induced by *K. pneumoniae*

As a type of extracellular vesicle, exosomes play a crucial role in intercellular communication [51]. Numerous studies have shown that exosomes derived from the serum of septic mouse models are widely involved in ALI through the regulation of miRNAs [52]. Macrophages not only act as carriers of exosomes but are also influenced by them [53, 54]. Dual-specific phosphatase 1 (DUSP1) plays a key role in dephosphorylating p38MAPK, thereby negatively regulating the p38MAPK pathway. Additionally, DUSP1 is positively regulated by mitogen- and stress-activated protein kinase-1 (MSK1) [55, 56]. To investigate the role of exosomes in ALI, a mouse model was established using iHvKp. Exosomes were then isolated from iHvKp-stimulated macrophages (ihvKp-exo). Notably, the expression of miR-155-5p in ihvKp-exo increased significantly in a time-dependent manner [20]. Further analysis revealed that exosomal miR-155-5p directly targeted MSK1, leading to the downregulation of DUSP1. The activation of the p38MAPK signaling pathway in resting macrophages highlighted the proinflammatory effects of exosome-derived miR-155-5p. The systemic non-specific inflammatory response observed in sepsis is believed to be associated with macrophage M1 polarization and the excessive secretion of inflammatory cytokines [57]. miR-155-5p plays a significant role in promoting M1 macrophage polarization and enhancing its proinflammatory functions, thereby exacerbating sepsis-associated ALI caused by *K. pneumoniae* infection. Conversely, the reduction of miR-155-5p levels led to decreased M1 polarization and alleviated inflammatory lung tissue damage [20]. Further experiments demonstrated that under iHvKp stimulation, miR-155-5p participates in the MSK1/DUSP1/p38MAPK signaling pathway, ultimately driving M1 macrophage polarization and inflammatory responses. In an animal model of pyoseptic pneumonia-associated ALI induced by iHvKp, inhibition of miR-155-5p resulted in improved lung tissue integrity and increased survival rates (see Table 1 for details). These findings suggest that targeting ihvKp-exo-induced miR-155-5p may offer a promising molecular approach for the treatment of iHvKp-associated ALI.

### Exosomal miR-181a-5p regulates the lung injury by *K. pneumoniae* infection

As a conserved miRNA, miR-181a-5p plays a crucial role in regulating pathological processes and is considered an important regulator of cancer [58]. It has also been linked to the development and function of NK cells [59] and contributes to the inflammatory response in conditions, such as pulmonary hypertension and chronic obstructive pulmonary disease [58, 60]. In a study on mice infected with Klebsiella pneumoniae, researchers found that the expression of adipose-derived mesenchymal stem cell (ADSC)-derived exosomal miR-181a-5p was upregulated in both BALF and BMDMs [17]. Signal transducer and activator of transcription 3 (STAT3) has been shown to play a significant role in *K. pneumoniae*-related injury [61, 62], and inhibiting STAT3 expression has been found to suppress the progression of *K. pneumoniae* infection [63]. Moreover, multiple studies have demonstrated that STAT3 is involved in activating the NLRP3 inflammasome [64], and its abnormal expression has been linked to several inflammatory diseases [65]. ADSC-derived exosomal miR-181a-5p mitigates *K. pneumoniae-*induced inflammation through a macrophage-related mechanism, reducing lung levels of IL-1β, IL-18, IL-8, TNF-α, and TGF-β. Further mechanistic studies have shown that miR-181a-5p targets STAT3 at the post-transcriptional level, thereby alleviating K*. pneumoniae*-induced lung injury [17] (see Table 1 for detailed mechanisms). Additionally, ASC has been identified as a key component of the inflammasome complex, mediating the secretion of inflammatory cytokines, such as IL-1β and IL-18 [66]. Therefore, further research is needed to explore the potential mechanisms of the miR-181a-5p/STAT3 pathway in *K. pneumoniae*-induced lung injury. These findings provide new insights into the inflammatory response and may contribute to the development of novel therapeutic strategies.

## The expression of miRNAs in sepsis-induced by *K. pneumoniae* infection

Sepsis is a life-threatening condition caused by organ dysfunction resulting from a dysregulated host response to bacterial infection [67]. *K. pneumoniae* is one of the most common pathogens responsible for sepsis [68]. Although the timely administration of antibiotics has reduced sepsis-related mortality, the death rate has remained high over the past few decades [69]. YgiM, originally identified as an intimal protein in *Escherichia coli* [70], has been found to localize in peroxisomes in both yeast and human cells [71]. A homologous gene (vk055_4013), highly similar to ygiM, has also been discovered in *K. pneumoniae*. Research suggests that the loss of ygiM enhances *K. pneumoniae* resistance to macrophage phagocytosis by targeting host cell peroxisomes, thereby improving the bacterium’s intracellular survival [69]. In a mouse model of *K. pneumoniae*-induced sepsis, differentially expressed miRNAs and their potential target mRNAs were identified. Among the ygiM-related miRNAs, miR-7108-5p, miR-4433b-3p, and miR-342-3p were highlighted for their novel association with sepsis [69]. The specific interaction networks of ygiM include miR-342-3p/VNN1, miR-7108-5p/CEACAM8, miR-4433b-3p/CEACAM8, and miR-342-3p/CEACAM8 [69]. These findings provide new insights into the role of YgiM and miRNAs in *K. pneumoniae*-induced sepsis. miR-155 is believed to play a significant regulatory role in the liver during *K. pneumoniae* sepsis. It has been implicated in the formation of neutrophil extracellular traps—an important immune defense mechanism against *K. pneumoniae* invasion [72, 73]. Additionally, myeloid miR-155 has been shown to exacerbate organ damage in *K. pneumoniae* sepsis [43]. These findings suggest that abnormally expressed miR-155 could serve as a novel biomarker for predicting mortality and treatment outcomes in severe sepsis [74, 75]. Moreover, they highlight the crucial role of miR-155 in the host defense response to *K. pneumoniae*-induced sepsis. This version corrects grammatical issues, improves sentence flow, and enhances clarity while maintaining the technical details.

## The expression of miRNAs in peritonitis induced by *K. pneumonia* infection

Peritonitis is typically classified into primary, secondary, and tertiary peritonitis [76]. Primary peritonitis includes spontaneous bacterial peritonitis and peritoneal dialysis-associated infections [77]. Patients with peritonitis remain at high risk of developing sepsis, which can lead to organ failure and death [77]. *Klebsiella spp*. is the second most common Gram-negative bacterium isolated from ICU peritonitis patients [78]. In a mouse peritonitis model infected with *K. pneumoniae*, eight miRNAs, including miR-21, were upregulated, while miR-375 was downregulated [79]. Mice that gradually regained weight following *K. pneumoniae* infection were classified into a survival group, whereas those with continued weight loss were classified into a non-survival (dead) group. Compared to normal mice, the survival group exhibited significant dysregulation of miR-203 and miR-672. Among the five upregulated miRNAs in the non-survival group (miR-21, miR-183, miR-301a, miR-652, and miR-672), only miR-301a showed a statistically significant difference. Furthermore, compared to the survival group, 18 miRNAs were differentially expressed in the non-survival group, with miR-672 showing lower expression, while the rest were upregulated [79]. TLR2 and TLR4 are key signaling molecules involved in recognizing *K. pneumoniae*, and their expression is upregulated during infection [80]. Several miRNAs, including miR-155-5p, miR-142-3p, and miR-23b-5p, are known to target key components of the TLR signaling pathway [81–83]. These findings suggest that during *K. pneumoniae*-induced peritonitis, differentially expressed miRNAs interact with the TLR pathway, providing insights into the infection mechanism (detailed in Table 1). In another peritonitis study [21], mice were pretreated with either saline or LPS before being infected with *K. pneumoniae*. Findings indicated that miR-155 downregulation in the LPS group was associated with significant suppression of TNF-α, likely contributing to *K. pneumoniae* tolerance through the targeting of SOCS1 and SHIP1 (inhibitors of the TLR pathway). Additionally, miR-146a was significantly upregulated, targeting key molecules in the TLR/IL-1 receptor pathway, such as IRAK1/2 and TNF receptor-associated factor 6 (TRAF6), ultimately inhibiting the inflammatory response [21]. Moreover, miR-132 and miR-21 were significantly upregulated in the LPS group and were predicted to target and inhibit IL-1β. Similarly, miR-142-3p was predicted to target and suppress IL-6, which may have further contributed to *K. pneumoniae* tolerance [21] (detailed in Table). In conclusion, the differential expression of multiple miRNAs in *K. pneumoniae* peritonitis modulates key inflammatory signaling pathways and significantly regulates pro-inflammatory cytokine levels, playing a crucial role in the disease progression.

## Reduction of SUMOylation via let-7 enhanced the process of *K. pneumoniae* infection

Small ubiquitin-like modifier (SUMO) proteins are a class of small ubiquitin-like proteins that serve as essential and widely used reversible post-translational protein modifiers. They play a key role in regulating infectious processes [84, 85]. Increased SUMOylation enhances the ability of host cells to combat *K. pneumoniae* infection [22]. Let-7 miRNAs, which can be regulated by type I interferon (IFN I) [86], are known tumor suppressors that target multiple oncogenes [87]. They also play a crucial regulatory role in inflammation [88]. Experimental results indicate that *K. pneumoniae*-infected macrophages induce IFN I production, which then signals through IFNAR1 to activate the expression of let-7 [22]. Upregulated let-7 inhibits SUMOylation, thereby promoting *K. pneumoniae* infection and limiting host inflammation. These findings suggest that *K. pneumoniae* infection triggers macrophages to utilize IFN I-induced let-7, leading to decreased SUMOylation as a pathogen-driven mechanism to suppress inflammation [22] (see Table 1 for detailed mechanisms). Additionally, it is suggested that let-7 plays a significant role in the host’s resistance to *K. pneumoniae* infection. Finally, Table 1 summarizes the functions and mechanisms of miRNAs in different types of *K. pneumoniae* infection.

## Conclusion

In recent decades, *K. pneumoniae* has become a major cause of both hospital- and community-acquired infections. The emergence of hvKP and MDR-KP has posed a significant threat to public health [89]. Currently, vaccines utilizing bacterial components [90] and incorporating advanced computational methods and artificial intelligence (AI) [91] offer promising strategies to prevent infections and reduce antimicrobial resistance.At the same time, it is crucial to conduct in-depth research on the changes in host cell biomolecules following *K. pneumoniae* infection. These biomolecules may help elucidate the mechanisms of bacterial infection. The 2024 Nobel Prize in Physiology or Medicine was awarded for research on miRNAs, which has undoubtedly inspired scientists to further investigate their role in disease occurrence, progression, and treatment. A review of the literature suggests that miRNAs play a key role in regulating gene expression and are involved in various infectious disease processes. This article summarizes the common clinical diseases caused by *K. pneumoniae* infections. Based on clinical research, laboratory animal models, and cellular studies, we have reviewed and preliminarily elucidated the functions and mechanisms of key miRNAs. Understanding the changes and effects of miRNAs in *K. pneumoniae* infections is of great significance, as it may provide new insights for treatment. However, the specific regulatory mechanisms of miRNAs in K. pneumoniae infections remain largely unclear, and their role in *K. pneumoniae*-host interactions requires further exploration. Advancing this research will contribute to the ongoing fight against bacterial infections.
